# The ratio of the seroprevalence to the egg-positive prevalence of *Schistosoma japonicum* in China: a meta-analysis

**DOI:** 10.1186/s12879-018-3320-5

**Published:** 2018-08-15

**Authors:** Yao Deng, Chen Qiu, Huan Ding, Da-Bing Lu

**Affiliations:** 1 Department of Epidemiology and Statistics, School of Public Health, Soochow University, Suzhou, China; 2 Jiangsu Key Laboratory of Preventive and Translational Medicine for Geriatric Diseases, School of Public Health, Soochow University, Suzhou, People’s Republic of China; 3 Key Laboratory of National Health and Family Planning Commission on Parasitic Disease Control and Prevention, Jiangsu Provincial Key Laboratory on Parasite and Vector Control Technology, Jiangsu Institute of Parasitic Diseases, Wuxi, Jiangsu Province China

**Keywords:** *Schistosoma japonicum*, Kato-Katz, IHA, ELISA, Prevalence ratio

## Abstract

**Background:**

Schistosomiasis, caused by *Schistosoma japonicum*, remains one of the most important parasitic diseases, and detection of *S. japonicum* infections in humans plays a crucial role in control and treatment. However, comparisons between the parasitological and the immunological examinations in the fields of China are lacking. Therefore we performed a meta-analysis to compare the seroprevalence of *Schistosoma japonicum*, as determined by IHA or ELISA, with coprological prevalence, as determined by Kato-Katz, and estimate the ratio of the serological to the egg-positive prevalence in order to evaluate the potential threat of egg-negative but worm-positive schistosomiasis.

**Methods:**

Studies published up to July 2018 on the parasitological and immunological examinations of schistosomiasis in the fields of China were searched in five databases including CNKI, WanFang, VIP, PubMed and Web of Science. The ratio of the serological to the egg-positive prevalence and its 95%CI for each study were calculated, and then point estimates and their 95%CIs of pooled prevalence ratio were meta-analyzed. Subgroup meta-analyses were also performed according to potential influential factors.

**Results:**

A total of 23 articles were included. The prevalence ratio varied from 0.57 to 48.83 for IHA to Kato-Katz and ranged from 0.38 to 13.97 for ELISA to Kato-Katz. The pooled ratio was 4.72 (95%CI: 3.87~ 5.76) for IHA to KK and 4.65 (95%CI: 3.50~ 6.17) for ELISA to KK. Subgroup analyses implied that the ratio of the serological to the egg-positive prevalence may decrease with the endemic levels. The highest prevalence ratio was observed when Kato-Katz was performed with three slides per stool or in hilly and mountainous regions.

**Conclusions:**

The worm-determined prevalence by IHA or ELISA is 4- to 5-fold higher than the egg-determined prevalence by Kato-Katz, which implied Kato-Katz may largely underestimate the prevalence of *S. japonicum* in China. The degree of underestimation was greater when Kato-Katz with three slides per stool was carried out, especially in low endemic areas or in hilly and mountainous regions. Therefore, more attention should be paid to those egg-negative but worm-positive patients with the aim of final elimination of *S. japonicum* in China.

**Electronic supplementary material:**

The online version of this article (10.1186/s12879-018-3320-5) contains supplementary material, which is available to authorized users.

## Background

Schistosomiasis, caused by blood-dwelling flukes, is one of the most prevalent parasitic diseases with over 200 million people infected, nearly 800 million people threatened and 70 million disability-adjusted life-years lost worldwide [1]. In China where *Schistosoma japonicum* was once highly endemic, after nearly 70 years of control a great progress has been made [2]. For example, the number of infected people had declined from 11.6 million in the mid-1950s to nearly 77.2 thousand in 2015 [3, 4]. By the end of 2015, out of 453 endemic counties (city, district), 343 and 110 had achieved criteria of transmission interruption and of transmission control, respectively. The overall objective of the Mid- and Long-term National Plan for Prevention and Control of Schistosomiasis of China (2004–2015) has been achieved on schedule [5]. Based on above achievements, therefore, a new goal-to interrupt transmission by 2020 and then to completely eliminate the disease across China by 2025-for the next 10 years was proposed in 2015 [6]. However, due to the complex lifecycle of *Schistosoma japonicum* and many natural and social factors in affecting the transmission and spread of schistosomiasis, schistosomiasis elimination still faces serious challenges. In addition, no effective vaccine is available and praziquantel is the only recommended drug by WHO for the treatment of schistosomiasis [7, 8]. Therefore, accurately assessing the real prevalence of *Schistosoma japonicum* infections in humans through simple and sensitive diagnostic tests is very essential in China [9].

There are currently various diagnostics for *S. japonicum* infection in humans, including direct parasitological tests, immunological techniques and molecular diagnosis [10]. Direct parasitological tests, such as the Kato-Katz thick smear technique (Kato-Katz or KK), are the earliest diagnosis used for identification of schistosomiasis and are still widely applied nowadays [11]. However, with the continual decrease of the prevalence and intensity of *S. japonicum* infection, parasitological tests are not sensitive and accurate enough to estimate the real prevalence, especially in low endemic regions [12–14]. Immunological techniques, including the indirect hemagglutination assay (IHA) and the enzyme-linked immunosorbent assay (ELISA), have become more popular in schistosome detection as they are more sensitive, rapid and easy to perform [15, 16]. A number of studies have been conducted to evaluate the effectiveness of different diagnostic assays either in the laboratory or in the field. However, the different or controversial results were among these findings. A meta-analysis on the immunodiagnostic efficacies of IHA and ELISA in field settings performed by Wang et al. [17] revealed that IHA is superior to ELISA, but the research by Wang et al. [18] showed that ELISA is better than IHA. Three other meta-analyses [19–21] were used to assess the accuracy of IHA, ELISA and the dipstick dye immunoassay (DDIA), respectively. All the above articles were mainly aimed at immunological tests. However, comparisons between parasitological and immunological examinations of the same village-based subjects in the field of China have not yet been conducted. The neglect of egg-negative but worm-positive schistosomiasis, especially in low endemic areas, may influence control effects and thus hinder the elimination of schistosomiasis [22]. Therefore, we performed this meta-analysis to compare the seroprevalence of *Schistosoma japonicum*, as determined by IHA or ELISA, with coprological prevalence, as assessed using Kato-Katz, and estimate the ratio of the serological to the egg-positive prevalence. The main purpose was to evaluate the potential threat of egg-negative but worm-positive schistosomiasis.

## Methods

### Study protocol and registration

The protocol for this study was developed prospectively and registered in the international Prospective Register of Systemic Reviews (PROSPERO) online database (https://www.crd.york.ac.uk/PROSPERO, with registered number CRD42017067941) on June 6, 2017. The protocol is provided as Supporting Information (see Additional file 1).

### Search strategy

A database search through July 2018 was performed to identify relevant studies regarding the comparison between seroprevalence of *S. japonicum* determined by IHA or ELISA and coprological prevalence with Kato-Katz. We aimed to include all published studies in Chinese or English. Three Chinese literature databases, including China National Knowledge Infrastructure (CNKI), WanFang Database and Chinese Scientific Journal Database (VIP), and two English literature databases, including PubMed and Web of Science, were searched for data pertaining to the prevalence rate of *S. japonicum* determined by three diagnostic tests in field surveys in China. We used the following search terms (the corresponding Chinese keyword in Pinyin (phoneticism) was given between brackets): “*schistosoma*”, “schistosomiasis”, “bilharzia”, “bilharziasis” (xuexichong) in combination with “Kato” and “IHA” or “ELISA”. We did not contact authors of original studies for additional information. No attempt was made to identify unpublished studies.

### Study selection

An initial screen of identified titles and abstracts was performed by YD and CQ. A second screen of full-text articles was then conducted if the studies were found suitable for inclusion. Studies to qualify the following criteria were included: (1) field surveys; (2) targeted subjects included residents from endemic villages, and received both a fecal examination with Kato-Katz and a serological examination with IHA or ELISA, simultaneously; (3) a survey in one village, or more villages of the same endemic level, could be taken as a study; (4) one stool sample from each participant was provided; (5) data on numbers of the positive and the total examined were provided, or could be obtained by formula. Studies to exclude were: (1) about animals or from laboratories; (2) in the same village and repeated periods; (3) no eggs found in a stool specimen; (4) about floating populations, such as fishermen and boatmen; (5) full texts unavailable.

### Data extraction and quality assessment

The detailed features of each eligible study were extracted using a purpose-built data-collection excel form. Information was recorded as follows: study characteristics (last name of the first author, year of publication, period of study, location); characteristics of targeted villages (the recorded prevalence level and the type of endemic area); study methodology (the fecal and serological examinations used); numbers of the positive and the total of persons assessed. Information about the endemic level or type of the targeted villages was extracted from relevant articles if such data were not provided in the eligible studies.

Bias in data collection was reduced through the involvement of YD and CQ, who independently evaluated the quality of studies. In cases of disagreement, HD was consulted in order to resolve the problem.

### Data analyses

The ratio of the serological to the egg-positive prevalence and its 95%CI for each study were first calculated. We then meta-analyzed the point estimate of pooled ratio and the corresponding 95%CI [23, 24].

In all analyses, Cochran’s Q test (significance level at *P <* 0.10) and I^2^ statistics were applied to measure the heterogeneity [25]. The I^2^ statistics is a quantitative measure of inconsistency across studies, with values of 25, 50 and 75% corresponding to low, moderate, and high degrees of heterogeneity, respectively [26]. In the absence of observed heterogeneity between studies, the fixed effects model should be used for the data analysis; otherwise, the random effects model should be selected [27, 28]. We conducted subgroup analyses stratified by the endemic level or the type of targeted villages, or the number of slides used per stool, to evaluate the influence of these factors on outcomes.

Potential publication bias of studies was assessed with both Begg rank correlation test and Egger linear regression test [29, 30]. To test the robustness of the pooled ratio, we conducted a sensitivity analysis by omitting one study or publication at a time, and each time we calculated the pooled estimate for the remaining ones [31].

Extracted data were entered into Microsoft Office Excel 2013 and all statistical analyses were performed with Stata 14.0. *P*<0.05 was considered statistically significant, except where otherwise specified. All statistical tests were two-sided. The PRISMA (Preferred Reporting Items for Systematic reviews and Meta-Analyses) statement was used as a guide in this paper [32].

## Results

### Literature search

**Fig. 1:**
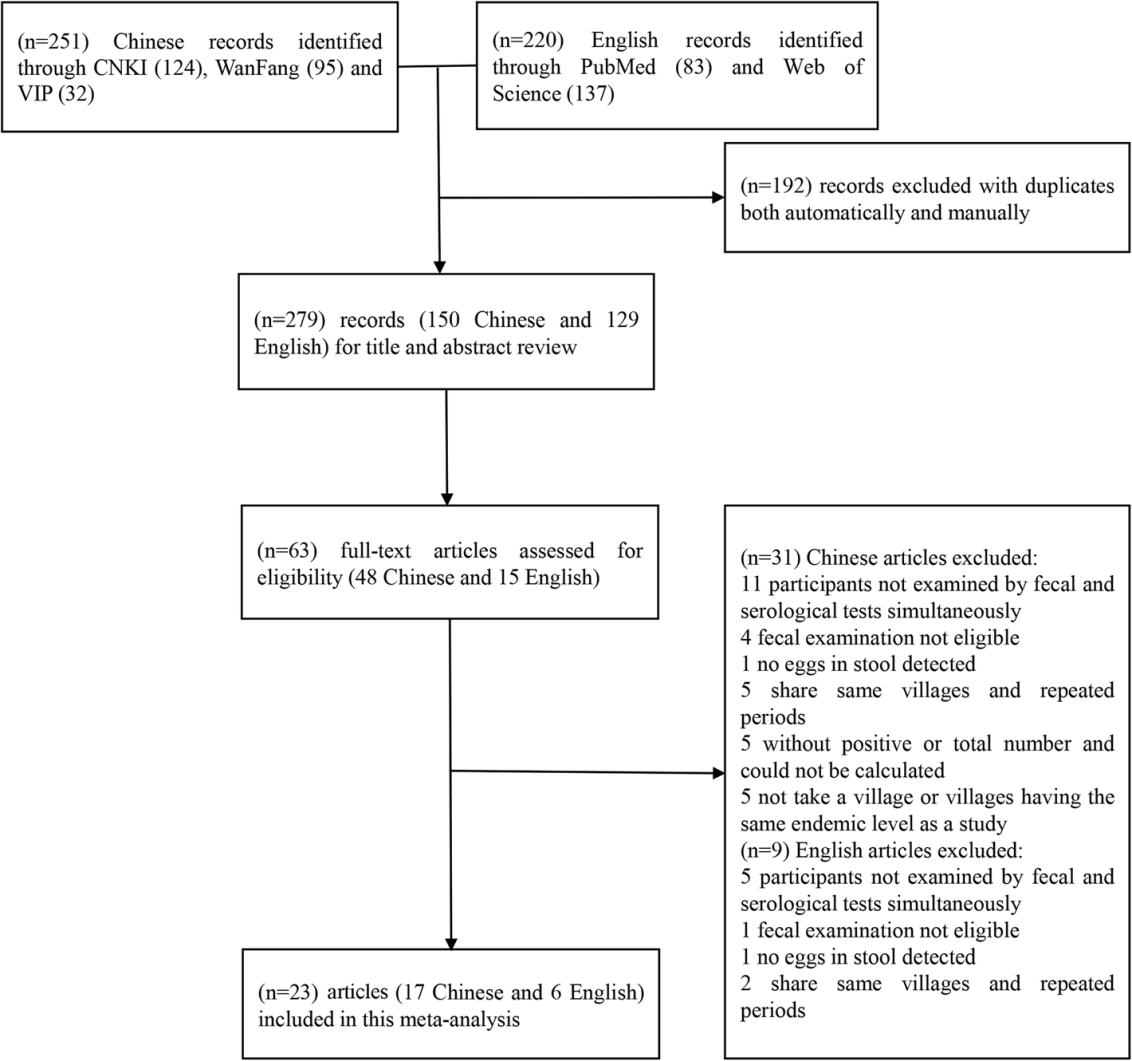
Flow chart of study selection

Figure 1 contains a flow chart that describes search results and selection strategy for the studies included in this meta-analysis. We retrieved 471 published articles through five databases, of which 192 articles were excluded when taking duplication into consideration. After the initial screening of titles and abstracts, a further 216 articles were excluded. Consequently, we identified 63 potentially relevant publications for full-text review, of which 40 articles were excluded according to the inclusion criteria. Finally, a total of 23 articles were enrolled in this meta-analysis, with 11 articles about IHA [33–44], six about ELISA [45–50] and six about both IHA and ELISA [51–55].

### Study characteristics

Table 1 and Table 2 show the characteristics of the final 23 publications, which covered six provinces, namely Jiangsu, Anhui, Hunan, Hubei, Jiangxi and Yunnan provinces. The years of the studies performed and published ranged from 1990 to 2012 and from 1992 to 2015, respectively. The number of participants ranged from 101 to 1956 with IHA, 68 to 1024 with ELISA and 68 to 1956 with Kato-Katz.

**Table 1:** Characteristics of the eligible studies with IHA and the Kato-Katz method

Author, year	Period of study	Location	Endemic level	Type of endemic area	Fecal slides per stool	Study group	No. of seropositive with IHA	No. total examined with IHA	No. of fecal positive	No. total examined with Kato-Katz	Prevalence ratio
Wu,1992 [51]	1990–1991	Gaojia village in Duchang County, Jiangxi	high (prevalence rate: 64.4% before treatment; 54.5% after treatment)	ML^a^	2	before treatment	110	125	33	125	3.33
						after treatment	102	125	3	125	34.00
Guan,1999 [33]	1997	Zhongjiang village in Baimasi Town, Hubei	high (20–30%)	PW^b^	2		442	707	193	774	2.51
Li,2002 [34]		Sanlian and Lianhe villages in Dangtu County, Anhui	low (2.23%)	ML	3		49	616	15	616	3.27
Xiao,2005 [36]	2003	Dongliang (village A) in Wuhu City, Puxi (village B) in Lanling County, Anhui	low (village A: <2%) high (village B: 15%)	PW	3	Dongliang	43	501	2	501	21.50
						Puxi	240	625	66	625	3.64
Jin,2005 [35]	2003	Chengde, Guanghui,Tiehu, Xianjin,Nanjiang and Xuguang villages in Tongling County, Anhui	high (Chengde and Guanghui: >10%) middle (Tiehu and Xianjin: 5–10%) low (Nanjiang and Xuguang: 1–5%)	ML	3	Chengde	134	309	62	309	2.16
						Guanghui	156	308	54	308	2.90
						Tiehu	88	307	27	307	3.26
						Xianjin	79	294	61	294	1.30
						Nanjiang	34	308	60	308	0.56
						Xuguang	25	305	41	305	0.61
Bao,2006 [37]	2005	Xitan, Tangnan, Hongtang, Pudong villages in Nanling County, Anhui	high (Xitan, Tangnan: TypeIvillage) middle (Hongtang, Pudong: Type IIvillage)	PW	2	TypeIvillage	158	693	54	693	2.93
						TypeIIvillage	117	620	13	620	9.00
Xu,2007 [52]	2005	Lianhu village in Poyang County,Chaipeng village in Duchang County,Fuyu village in Yugan County, Jiangxi	high (Lianhu: 12.0%) middle (Chaipeng: 6.2%; Fuyu: 8.0%)	ML	3	Lianhu,IHA-A	706	873	83	961	9.36
						Chaipeng,IHA-A	147	243	25	401	9.70
						Fuyu,IHA-A	181	443	32	502	6.41
						Lianhu,IHA-B	390	873	83	961	5.17
						Chaipeng,IHA-B	90	243	25	401	5.94
						Fuyu,IHA-B	145	443	32	502	5.13
Yu,2007 [38]		Zhonjiang village in Hubei; Zhuxi village in Jiangxi		PW (Zhonjiang) ML (Zhuxi)	2	Zhonjiang	364	571	188	770	2.61
						Zhuxi	206	289	166	356	1.53
Zhou,2007 [53]		Village A in Jiangxi; Village B in Anhui	high (Village A: >10%) low (Village B: <5%)		3	Village A	528	1024	130	1024	4.06
						Village B	288	787	36	787	8.00
Zhou,2008 [40]	2001–2006	An administrative village in Jiangxi			3	2001	100	345	28	900	9.32
						2002	165	600	34	600	4.82
						2003	174	600	41	600	4.26
						2004	187	600	52	600	3.59
						2005	332	785	97	677	2.95
						2006	169	603	52	632	3.41
He,2008 [54]	2004	Fengyi village in Zongyang County; Linye village in Anqing City, Anhui	middle	ML	3	Fengyi	151	807	25	807	6.04
						Linye	25	216	2	216	12.50
Lin,2008 [12]		Xinhua village in Xingzi County, Jiangxi	middle (TypeIvillage)	ML	6		106	633	43	633	2.47
Zhong,2009 [41]	2008	Yanhu village in Xinjian County, Jiangxi	middle (5–10%)	ML	3		112	420	26	420	4.31
Hu,2010 [42]	2001	Changjiang village in Yueyang City, Hunan	low (3–5%)	ML	3		124	511	20	511	6.20
Lin,2010 [43]	2009	Villages in Huangzhou, Songzi and Xiaonan District, Hubei	low (Huangzhou: <1%; Songzi: 1–5%) middle (Xiaonan: 5–10%)	ML	3	Huangzhou	5	592	3	592	1.67
						Songzi	166	1296	9	1296	18.44
						Xiaonan	25	101	2	101	12.50
Lin,2010 [55]		Caohui village in Xinjian County, Jingtou village in Duchang County, Xinhua village in Xingzi County, Jiangxi		ML	3	Caohui,M4	475	883	55	883	8.64
						Caohui,M5	503	883	55	883	9.15
						Jingtou,M4	465	927	73	927	6.37
						Jingtou,M5	476	927	73	927	6.52
						Xinhua,M4	350	922	40	922	8.75
						Xinhua,M5	358	922	40	922	8.95
Liu,2014 [44]	2011	Yongle and Xinzhuang villages in Eryuan County, Yunnan	low (<1%)	HM^c^	3	Yongle	293	1956	6	1956	50.00
						Xinzhuang	58	778	11	778	5.36

^a^Marshlands and lakes region

^b^Plains with water networks

^c^Hilly and mountainous region

**Table 2:** Characteristics of the eligible studies with ELISA and the Kato-Katz method

Author, year	Period of study	Location	Endemic level	Type of endemic area	Type of ELISA	Fecal slides per stool	Study group	No. of seropositive with ELISA	No. total examined with ELISA	No. of fecal positive	No. total examined with Kato-Katz	Prevalence ratio
Wu,1992 [51]	1990–1991	Gaojiao village in Duchang County, Jiangxi	high (prevalence rate: 64.4% before treatment; 54.5% after treatment)	ML^a^	McAb-Dot -ELISA	2	before treatment	55	125	33	125	1.67
							after treatment	21	125	3	125	7.00
Huang,1994[45]	1990–1991	Lianshi,Xingou,Yangjiayuan,third and fifth group of Zhaonao villages in Qianjiang City, Hubei	low (Lianshi: <5%) middle (Xingou: 10%) high (Yangjiayuan and Zhaonao: 30%)	ML	Dot-ELISA	3	Lianshi	3	68	8	68	0.38
							Xingou	15	79	23	79	0.65
							Yangjiayuan	18	85	24	85	0.75
							Zhaonao III	24	68	24	68	1.00
							Zhaonao IV	46	109	15	109	3.07
Song,2003 [46]	2002	Tanzhu village in Gaozi Town, Jiangsu	low	ML	SEA-ELISA	3		85	463	9	463	9.44
Bao,2006 [37]	2005	Xitan, Tangnan, Hongtang, Pudong four villages in Nanling County, Anhui	high (Xitan, Tangnan: TypeIvillage) middle (Hongtang, Pudong: Type IIvillage)	PW^b^	SEA-ELISA	2	GradeItype village	70	324	54	693	2.77
							GradeIItype village	71	303	13	620	11.18
He,2007		Shujie village in Weishan County,Yunnan	high	HM^c^	SEA-ELISA	3		423	508	120	508	3.53
Chen,2007	2005	Three villages in Eryuan County,Yunnan	low, middle and high endemic village respectively	HM	SEA-ELISA	4	low epidemic	22	107	2	107	11.00
							middle endemic	67	128	7	128	9.57
							high epidemic	67	116	6	116	11.17
Xu,2007	2005	Lianhu village in Poyang County,Chaipeng vliiage in Duchang County,Fuyu village in Yugan County, Jiangxi	high (Lianhu: 12.0%) middle (Chaipeng: 6.2%; Fuyu: 8.0%)	ML	F-ELISA	3	Lianhu	446	873	83	961	5.92
							Chaipeng	130	243	25	401	8.58
							Fuyu	183	443	32	502	6.48
Zhou,2007		Village A in Jiangxi; Village B in Anhui	high (Village A: >10%) low (Village B: <5%)		SEA-ELISA	3	Village A	682	1024	130	1024	5.25
							Village B	503	787	36	787	13.97
He,2008	2004	Linye village in Anqing City, Anhui	middle	ML	SEA-ELISA	3	Linye	27	216	2	216	13.50
Lin,2010		Caohui village in Xinjian County,Jingtou vliiage in Duchang County,Xinhua village in Xingzi County, Jiangxi		ML	SEA-ELISA	3	Caohui,M3	504	883	55	883	9.16
							Jingtou,M3	358	927	73	927	4.90
							Xinhua,M3	355	922	40	922	8.88
Yu,2011		Village A in Jiangxi			SEA-ELISA	3		80	333	13	333	6.15
She,2015	2012	Dahekou village in Donggou Town, Jiangsu	low (reach transmission control status in 2009)	ML	SEA-ELISA	3		19	583	2	583	9.50

^a^Marshlands and lakes region

^b^Plains with water networks

^c^Hilly and mountainous region

### Main analysis

**Fig. 2:**
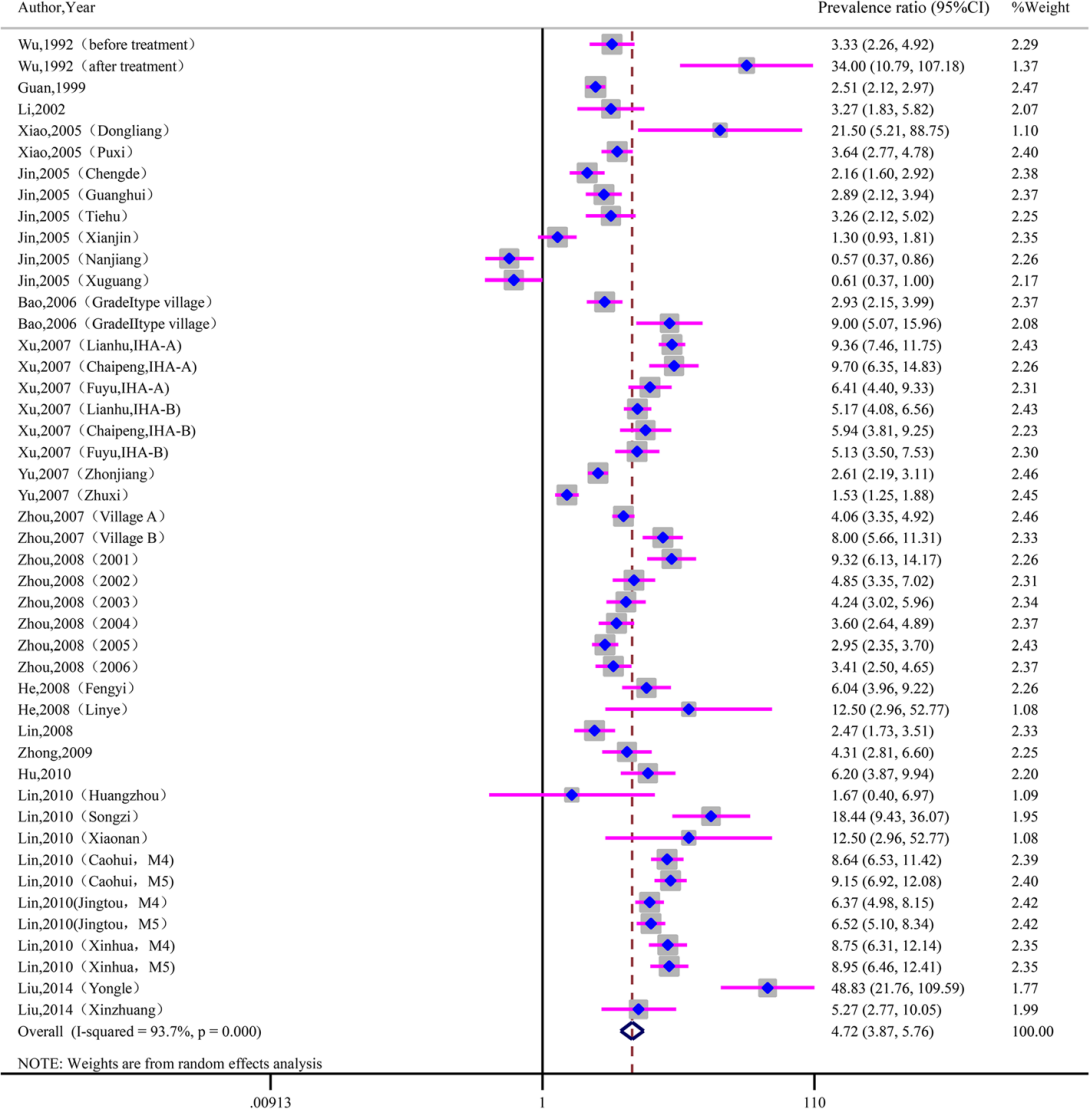
Forest plot of prevalence ratio for IHA to Kato-Katz with random-effects analyses

**Fig. 3:**
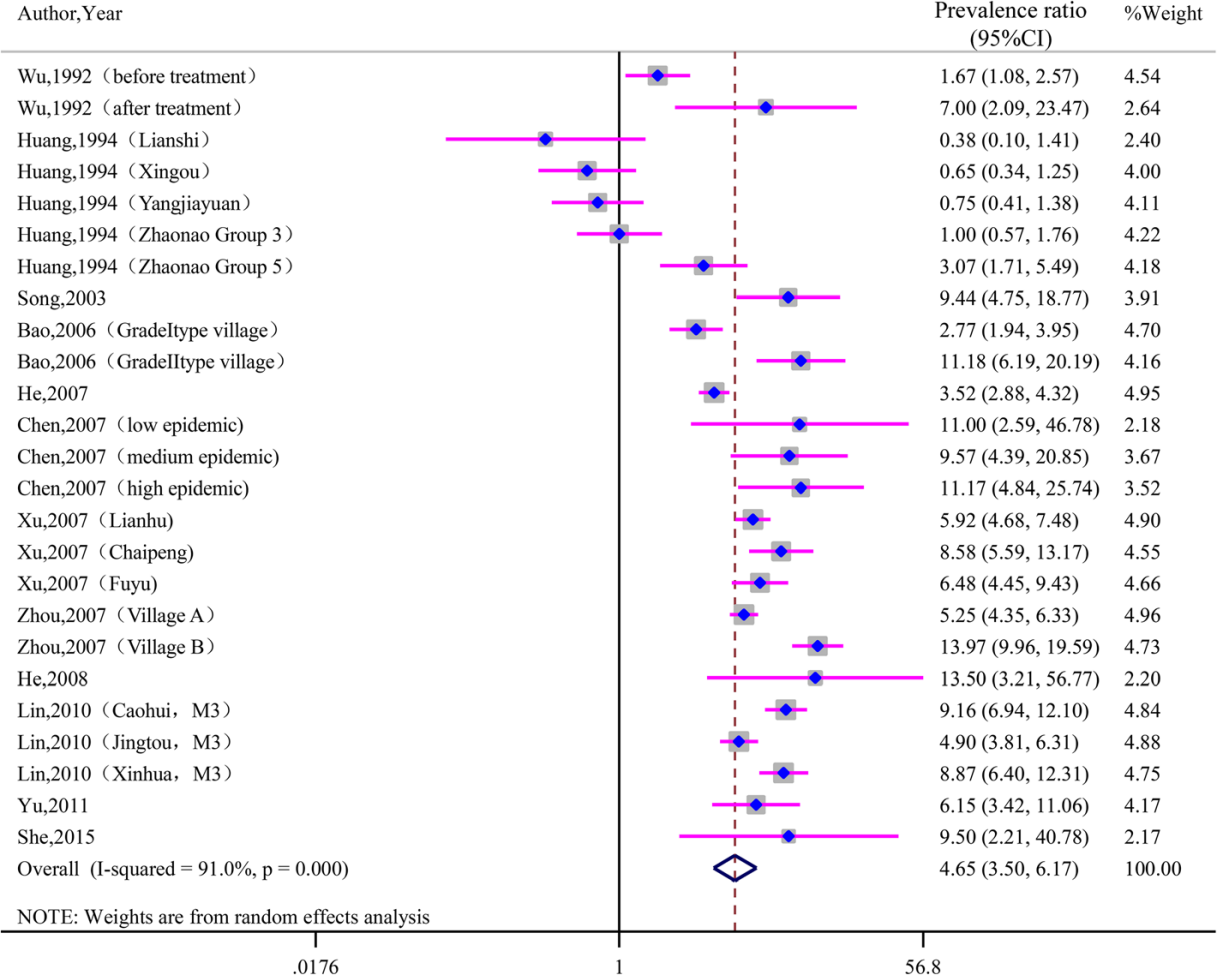
Forest plot of prevalence ratio for ELISA to Kato-Katz with random-effects analyses

As seen in Fig. 2, the prevalence ratio of IHA to Kato-Katz varied from 0.57 to 48.83, with substantial heterogeneity among studies (χ^2^ = 713.34, *P* < 0.001; I^2^ = 93.7%). The prevalence ratio of ELISA to Kato-Katz ranged from 0.38 to 13.97, with substantial heterogeneity among studies (χ^2^ = 266.77, *P* < 0.001; I^2^ = 91.0%). See Fig. 3. The pooled prevalence ratio was 4.72 (95%CI: 3.87~ 5.76) for IHA to Kato-Katz and 4.65 (95%CI: 3.50~ 6.17) for ELISA to Kato-Katz when calculated with the random-effects model.

### Subgroup analyses

Table 3 shows the results of subgroup analyses stratified by the endemic level, the number of slides used per stool sample and the type of endemic area. Due to the high heterogeneity among studies within most subgroups, pooled ratios for each subgroup were calculated using the random-effects model. In terms of endemic levels, the pooled ratio of IHA to Kato-Katz showed the highest estimate in the middle endemic areas (RR: 4.99, 95%CI: 3.39~ 7.34) and the lowest in high endemic (RR: 4.05, 95%CI: 2.93~ 5.60). A downtrend was observed in the pooled ratios of ELISA to Kato-Katz (Low endemic areas: 6.06, 95%CI: 2.22~ 16.58; Middle: 5.92, 95%CI: 2.74~ 12.80; High: 3.06, 95%CI: 2.09~ 4.50). In terms of the number of slides per stool in Kato-Katz, the highest ratio of IHA to Kato-Katz (PRR: 5.03, 95%CI: 4.05~ 6.25) was obtained for three slides per stool specimen, whereas the lowest ratio (PRR: 2.47, 95%CI: 1.70~ 3.57) was obtained for over four slides per stool. The ratio of ELISA to Kato-Katz increased with the number of slides used (2 slides: 4.11, 95%CI: 1.76~ 9.61; 3 slides: 4.41, 95%CI: 3.20~ 6.08; ≥4 slides: 10.38, 95%CI: 5.97~ 18.05).

**Table 3:** Pooled estimates of prevalence ratio by potential influential factors with meta-analysis

Group	IHA to KK					ELISA to KK				
	No. of papers	No. of studies	Prevalence ratio (95%CI)	*P*_heterogeneity_	I^2^(%)	No. of papers	No. of studies	Prevalence ratio (95%CI)	*P*_heterogeneity_	I^2^(%)
Total	17	46	4.72(3.87,5.76)	0.000	93.7	12	25	4.65(3.50,6.17)	0.000	91.0
Endemic level										
Low	7	10	4.89(1.92,12.43)	0.000	95.9	5	5	6.06(2.22,16.58)	0.000	84.2
Middle	7	12	4.99(3.39,7.34)	0.000	86.6	5	6	5.92(2.74,12.80)	0.000	89.0
High	7	10	4.05(2.93,5.60)	0.000	91.5	7	10	3.06(2.09,4.50)	0.000	88.7
Number of slides per stool										
2	4	7	3.45(2.37,5.03)	0.000	89.2	2	4	4.11(1.76,9.61)	0.000	88.2
3	12	38	5.03(4.05,6.25)	0.000	91.7	9	18	4.41(3.20,6.08)	0.000	90.6
≥ 4	1	1	2.47(1.70,3.57)	–	–	1	3	10.38(5.97,18.05)	0.965	0.0
Type of endemic area										
Marshlands and lakes region	11	30	4.60(3.45,6.14)	0.000	93.8	7	16	3.73(2.48,5.60)	0.000	90.7
Plains with water networks	4	6	3.64(2.64,5.00)	0.000	82.2	1	2	5.45(1.37,21.66)	0.000	93.3
Hilly and mountainous regions	1	2	15.86(1.47,171.60)	0.000	95.2	2	4	7.20(3.33,15.57)	0.005	76.5

Subgroup analyses showed the highest pooled estimates in the hilly and mountainous regions (IHA to KK: 15.86, 95%CI: 1.47~ 171.60; ELISA to KK: 7.20, 95%CI: 3.33~ 15.57). The lowest was in plains with water network areas for IHA to Kato-Katz (RR: 3.64, 95%CI: 2.64~ 5.00) and in marshlands and lake regions for ELISA to Kato-Katz (RR: 3.73, 95%CI: 2.48~ 5.60).

### Sensitivity analyses

Sensitivity analyses presented that all single-study-omitted and single-paper-omitted estimates were within the 95%CIs of their respective overall ratios except one paper (see Additional files 2, 3, 4 and 5). This suggested that the pooled ratios were not substantially modified by any single study, or by any single paper except Huang et al. [45]. The stability of such results validated the rationality and reliability of our analyses.

### Publication bias

There was no evidence of publication bias, as suggested by Begg rank correlation test (z = 1.18, *P* = 0.237 for IHA and z = 0.54, *P* = 0.591 for ELISA) and Egger linear regression test (the bias coefficients b = 2.64, 95%CI: -0.17~ 5.46, *t* = 1.89, *P* = 0.065 for IHA and b = − 0.69, 95%CI: -3.45~ 2.07, *t* = − 0.52, *P* = 0.610 for ELISA).

## Discussion

Egg-negative but worm-positive schistosomiasis may be a potential threat for achieving the goal of schistosomiasis elimination [22]. Therefore, accurate detection of *Schistosoma japonicum* infections in humans plays a crucial role in the process of control and treatment [56]. There were also a number of other publications exploring the differences of parasitological and immunological diagnostic assays in field situations in China, but in those research Kato-Katz was only applied to human individuals after they had been tested positive with immunodiagnostic method, not qualifing the criteria of inclusion in our research [13, 56–69]. We here included only 23 articles in the current meta-analysis [33–55]. The findings of this meta-analysis showed that the prevalence by IHA or ELISA is 4- to 5-fold higher than the prevalence by Kato-Katz, which indicated that Kato-Katz clearly underestimated the prevalence of *S. japonicum* in China. Subgroup analyses suggested that the ratio of the serological to the egg-positive prevalence decreased with the endemic levels. A highest prevalence ratio was obtained when the number of slides per stool was three and was in the hilly and mountainous regions.

Compared with the egg-determined prevalence by a single stool examination, the worm-determined prevalence by IHA or ELISA were about 4–5 times higher, indicating the existence of substantial inconsistencies between two types of examinantions. Multiple stool examinations with Kato-Katz can increase the positive rate and obtain more accurate results, but it is impractical in the field [10, 70]. The Kato-Katz test is most widely used and the misdiagnosis or even absence of eggs can be quite common in the field, especially in low endemic regions [40, 71, 72]. Therefore, it is obvious that the positive prevalence with one stool examination is much lower than with IHA and ELISA.

Although the overall seroprevalence was about 4- to 5-fold higher than the pooled coprological prevalence, the prevalence ratio per study varied from 0.57 to 48.83 for IHA to KK and ranged from 0.38 to 13.97 for ELISA to KK. Several explanations could account for the substantial heterogeneity among studies. First, 14 eligible publications provided more than one group of studies and these studies could not be combined directly [35–38, 40, 43–45, 47, 51–55]. Second, study regions and periods varied in epidemiological settings, such as types of endemic areas, recorded prevalence level and transmission intensity. Third, different diagnostic reagents and the number of slides per stool may also cause the discrepancies. Fourth, sample sizes among included studies differed greatly, ranging from 68 to 1956. Finally, there are several cases which should be noted. The prevalence ratio of IHA to Kato-Katz was 50 in Yongle Village [44] and 34 after mass chemotherapy in Gaojia Village [51], which were much higher than the others. The infection rate in Yongle village was very low (0.8%) with Kato-Katz, leading to an underestimation of true infections [44]. In Gaojia village after 3 months of treatment the number of infections with Kato-Katz may decrease sharply but the antigen level of IHA in serum should be still very high, thus resulting in the high ratio [51]. When comparing Kato-Katz and ELISA, most articles applied the routine ELISA method to detect the antibodies, whereas two articles [45, 51] applied the Dot-ELISA method to detect the CAg in serum. However, the results between two papers were controversial. All these factors may lead to such differences among these studies.

Subgroup analyses, based on limited numbers of papers, revealed that the prevalence ratio decreased with the increase level of endemic degrees, although the ratio of IHA to ELISA was slightly higher in middle endemic villages than in low endemic villages. This may be mainly related to a high false negative rate for egg detection in stool in low endemic areas, particularly after widespread chemotherapy [73, 74].

Repeated and multiple stool examinations can provide more accurate results, but it is quite time-consuming and strenuous in the filed [10, 70]. As different stool specimens can not be compared directly, we only included articles in which a single stool examination was carried out. The findings showed that the highest prevalence ratio of IHA to Kato-Katz was 5.03 (95%CI: 4.05~ 6.25) with three slides per stool, and of ELISA to Kato-Katz was 10.38 (95%CI: 5.97~ 18.05) with ≥4 slides per stool. However, it is noted that only one article [47] provided data of fecal examinations with equal or more than four slides per stool specimen. So the uptrend of the prevalence ratio of ELISA to Kato-Katz with the increase of numbers of slides per stool was suspected. A relative high ratio was observed when examined with Kato-Katz with three slides per stool in our meta-analysis. Indeed, Lin et al. [75] once reported that the rates of underestimation with three slides per stool specimen could reach 40.98~ 50.80%, especially in low endemic areas.

There were only three articles involved in hilly and mountainous regions [44, 47, 48] with one on IHA to Kato-Katz and two on ELISA to Kato-Katz. We believed that the patients in Yongle village could, because of the low infection rate by the Kato-Katz method, be largely missed [44]. Overall, the current meta-analysis showed the highest prevalence ratio in hilly and mountainous regions. This may provide support for further schistosomiasis control among different endemic areas.

However, there are some disadvantages for immunological tests. The main one is the relatively high false-positive rate, as they can not discriminate active infection from past infection [76–78]. The false-positive rates of IHA and ELISA were reported to be 0.062 to 0.643 and 0.157 to 0.755, respectively [17]. In this meta-analysis, two articles [45, 51] applied the Dot-ELISA method to detect the CAg in serum. The negative reversal rate of egg-positive patients was 66.7% with McAb-Dot-ELISA and 0 with IHA after 3 months of treatment [51]. However, research also showed that most patients turned negative with IHA after effective and periodical treatment for 3 years or more [16]. After one to 2 years of treatment, the negative reversal rate was nearly 60% in the ELISA assay, and most patients turned negative in the Dot-ELISA test [16]. Overall, the Dot-ELISA assay appears to be more efficient than the routine IHA or ELISA. The false-positive rate of immunological tests is also a limitation of this paper, partly leading to an inflated prevalence ratio of IHA to Kato-Katz or of ELISA to Kato-Katz.

## Conclusions

In summary, the worm-determined prevalence by IHA or ELISA was about 4- to 5-fold higher than the egg-determined prevalence by Kato-Katz, which implied that the Kato-Katz method may largely underestimate the infection prevalence of *S. japonicum* in the field. The degree of underestimation became worse when being examined by Kato-Katz with three slides per stool, especially in low endemic areas or in hilly and mountainous regions. Such significant difference between the fecal and the serological examinations may lead to the existence of a sizable population of “egg-negative but worm-positive schistosomiasis”. Therefore, more attention should be paid to those infected humans with the aim of final elimination of *S. japonicum* in China.

## Additional files

Additional file 1: PROSPERO (CRD42017067941): A meta-analysis of the ratio of seroprevalence to egg-positive prevalence of *Schistosoma japonicum* in China. (PDF 117 kb)

Additional file 2: Sensitivity analyses for IHA to Kato-Katz by single-study-omitted. (TIF 447 kb)

Additional file 3: Sensitivity analyses for ELISA to Kato-Katz by single-study-omitted. (TIF 356 kb)

Additional file 4: Sensitivity analyses for IHA to Kato-Katz by single-paper-omitted. (TIF 247 kb)

Additional file 5: Sensitivity analyses for ELISA to Kato-Katz by single-paper-omitted. (TIF 214 kb)

## Abbreviations

CI: Confidence intervals
CNKI: China National Knowledge Infrastructure
DDIA: Dipstick dye immunoassay
ELISA: Enzyme-linked immunosorbent assay
IHA: Indirect hemagglutination assay
KK (Kato-Katz): Kato-Katz thick smear technique or the Kato-Katz method
PRISMA: Preferred reporting items for systematic reviews and meta-analyses
VIP: Chinese Scientific Journal Database
