# Supplementary material for: The ratio of the seroprevalence to the egg-positive prevalence of Schistosoma japonicum in China: a meta-analysis
Source: BMC Infect Dis. 2018 Aug 15;18:404. doi: 10.1186/s12879-018-3320-5 (PMC6094899; doi:10.1186/s12879-018-3320-5)
Supplement: Supplementary file 1 — PROSPERO (CRD42017067941): A meta-analysis of the ratio of seroprevalence to egg-positive prevalence of Schistosoma japonicum in China. (PDF 117 kb) [file 12879_2018_3320_MOESM1_ESM.pdf]

A meta-analysis of the ratio of seroprevalence to egg-positive prevalence of Schistosoma japonicum in China

Yao Deng

Citation

Yao Deng. A meta-analysis of the ratio of seroprevalence to egg-positive prevalence of Schistosoma japonicum in China. PROSPERO 2017:CRD42017067941 Available from
http://www.crd.york.ac.uk/PROSPERO_REBRANDING/display_record.asp?ID=CRD42017067941

Review question(s)

To perform a meta-analysis to compare the seroprevalence of Schistosoma japonicum, as determined by IHA or ELISA, with coprological prevalence, as assessed using the Kato-Katz method.

To estimate the ratio of serological to egg-positive prevalence, in order to evaluate the potential threat of egg-negative but worm-positive schistosomiasis.

Searches

The following electronic databases will be searched: PubMed, EMBASE, The Cochrane Library, Chinese National Knowledge Infrastructure (CNKI), Wanfang and VIP databases.

The search terms will include: (Schistosoma OR Schistosomiasis) AND Kato-Katz AND (IHA OR ELISA).

We will focus on literature published in English or Chinese, and there will be no restrictions imposed on publication period.

Types of study to be included

Field surveys.

Condition or domain being studied

Schistosomiasis, caused by Schistosoma japonicum, remains one of the most prevalent parasitic diseases, and has adverse impacts on public health and socioeconomic development in China, although great successes have been achieved after nearly 70 years of control.

Detection of schistosome infections in humans plays a crucial role in control and treatment. Three diagnostic methods have been consistently widely applied, namely the indirect hemagglutination assay (IHA), the enzyme-linked immunosorbent assay (ELISA), and quantitative Kato-Katz thick smear technique (the Kato-Katz method). This review aims to compare the positive rates of detection using fecal examination and serological examination techniques.

Participants/ population

Residents from endemic villages in China, who received fecal examinations (the Kato-Katz method) and serological examinations (IHA or ELISA) simultaneously, will be eligible for inclusion.

Studies on animals and from laboratories will not be included.

Intervention(s), exposure(s)

None.

Comparator(s)/ control

Seroprevalence estimates of Schistosoma japonicum as determined by IHA or ELISA will be compared with egg-

positive prevalence estimates as determined by the Kato-Katz technique in each targeted village.

Context
Schistosomiasis is more prevalent than previously thought, and Schistosoma japonicum is endemic in China. As serological assays for schistiosome detection are easy to perform and have been more widely used, we will perform a meta-analysis to compare the seroprevalence of Schistosoma japonicum, as determined by IHA or ELISA, with coprological prevalence, as assessed using the Kato-Katz method, and will estimate the ratio of serological to egg-positive prevalence in order to evaluate the potential threat of egg-negative but worm-positive schistosomiasis.

Outcome(s)
Primary outcomes
The ratio of seroprevalence, as determined by IHA or ELISA, to egg-positive prevalence, as indicated by the Kato-Katz method, of Schistosoma japonicum.

Secondary outcomes
The effects of various factors such as infection prevalence, the study year, any previous mass treatment regimes, etc. on the estimates obtained will also be investigated.

Risk of bias (quality) assessment
Bias in data collection will be reduced through the involvement of two reviewers, who will independently evaluate the quality of studies. In cases of disagreement, a third reviewer will be consulted in order to resolve the problem.

Publication bias will be minimized using funnel plots and Egger's correlation statistic.

Strategy for data synthesis
A narrative synthesis of the results of the study is planned. A between-study heterogeneity analysis will also be performed using the I-squared statistic. In the absence of observed heterogeneity between studies, the fixed effects model will be used for the analysis of the data, otherwise, the random effects model will be selected.

Analysis of subgroups or subsets
We will perform subgroup analyses based on the prevalence level, the study year, the study region, and any historical mass treatment regimes, if the data proves to be available.

Contact details for further information
Yao Deng

School of Public Health, Soochow University

199 Renai Road, Suzhou Industrial Park, Suzhou, Jiangsu, China 215123

1665286019@qq.com

Organisational affiliation of the review
Department of Epidemiology and Statistics, School of Public Health, Soochow University

http://gwxy.suda.edu.cn/

Review team
Miss Yao Deng,

Anticipated or actual start date
06 May 2017

Anticipated completion date
06 May 2018

Funding sources/sponsors
The National Science Foundation of China (to Da-Bing Lu, No.81273141)

Conflicts of interest
None known

Language
English

Country
China

Subject index terms status
Subject indexing assigned by CRD

Subject index terms
Biological Assay; China; Enzyme-Linked Immunosorbent Assay; Feces; Hemagglutination Tests; Humans; Parasite Egg Count; Prevalence; Schistosoma japonicum; Schistosomiasis japonica; Seroepidemiologic Studies

Stage of review
Ongoing

Date of registration in PROSPERO
06 June 2017

Date of publication of this revision
06 June 2017

Stage of review at time of this submission	Started	Completed
Preliminary searches	Yes	Yes
Piloting of the study selection process	Yes	Yes
Formal screening of search results against eligibility criteria	Yes	No
Data extraction	No	No
Risk of bias (quality) assessment	No	No
Data analysis	No	No

PROSPERO
International prospective register of systematic reviews
The information in this record has been provided by the named contact for this review. CRD has accepted this information in good faith and registered the review in PROSPERO. CRD bears no responsibility or liability for the content of this registration record, any associated files or external websites.
