# Supplementary figures and images for: The ratio of the seroprevalence to the egg-positive prevalence of Schistosoma japonicum in China: a meta-analysis
Source: BMC Infect Dis. 2018 Aug 15;18:404. doi: 10.1186/s12879-018-3320-5 (PMC6094899; doi:10.1186/s12879-018-3320-5)

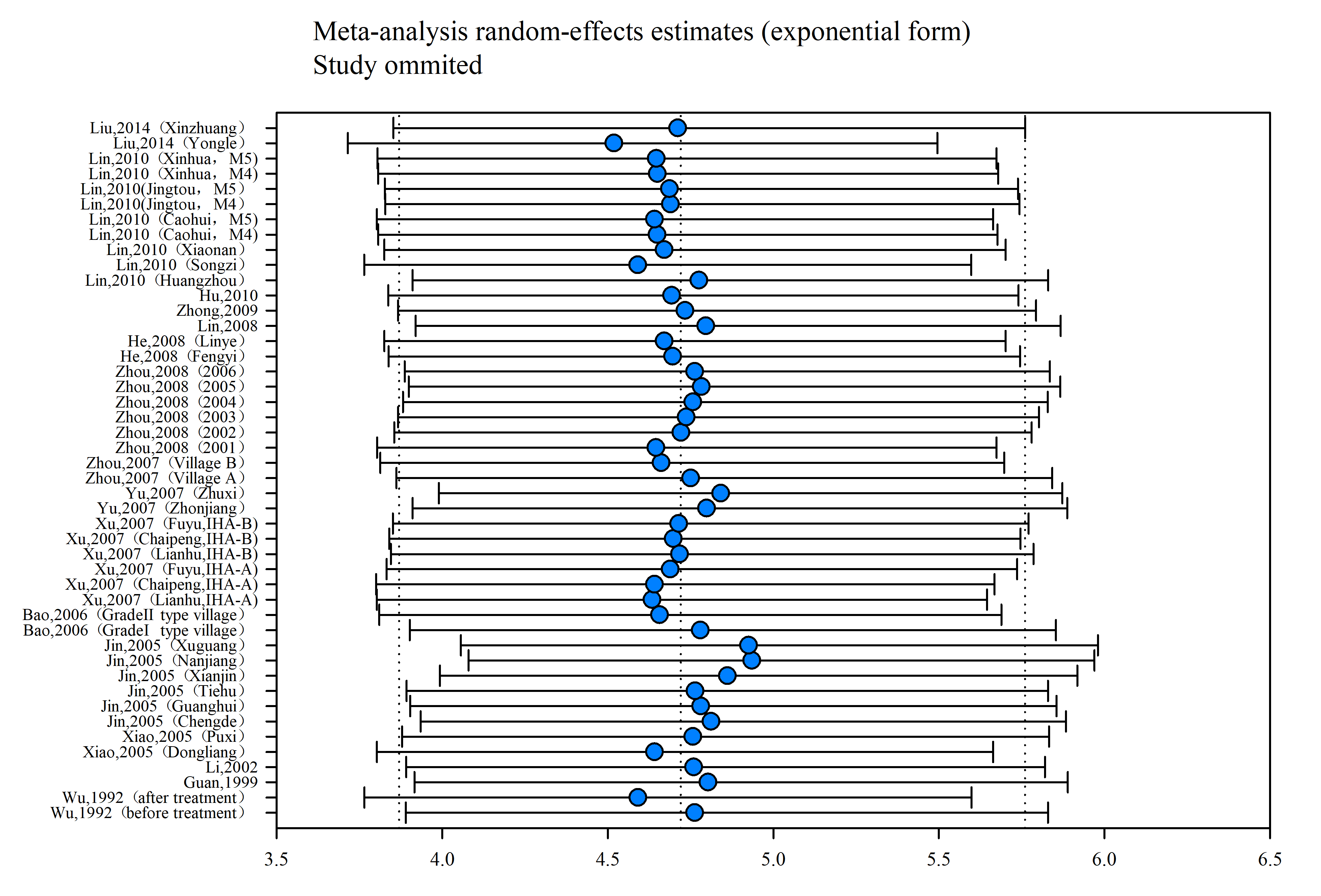

Supplement: Supplementary file 2 — Sensitivity analyses for IHA to Kato-Katz by single-study-omitted. (TIF 447 kb) [file 12879_2018_3320_MOESM2_ESM.tif]

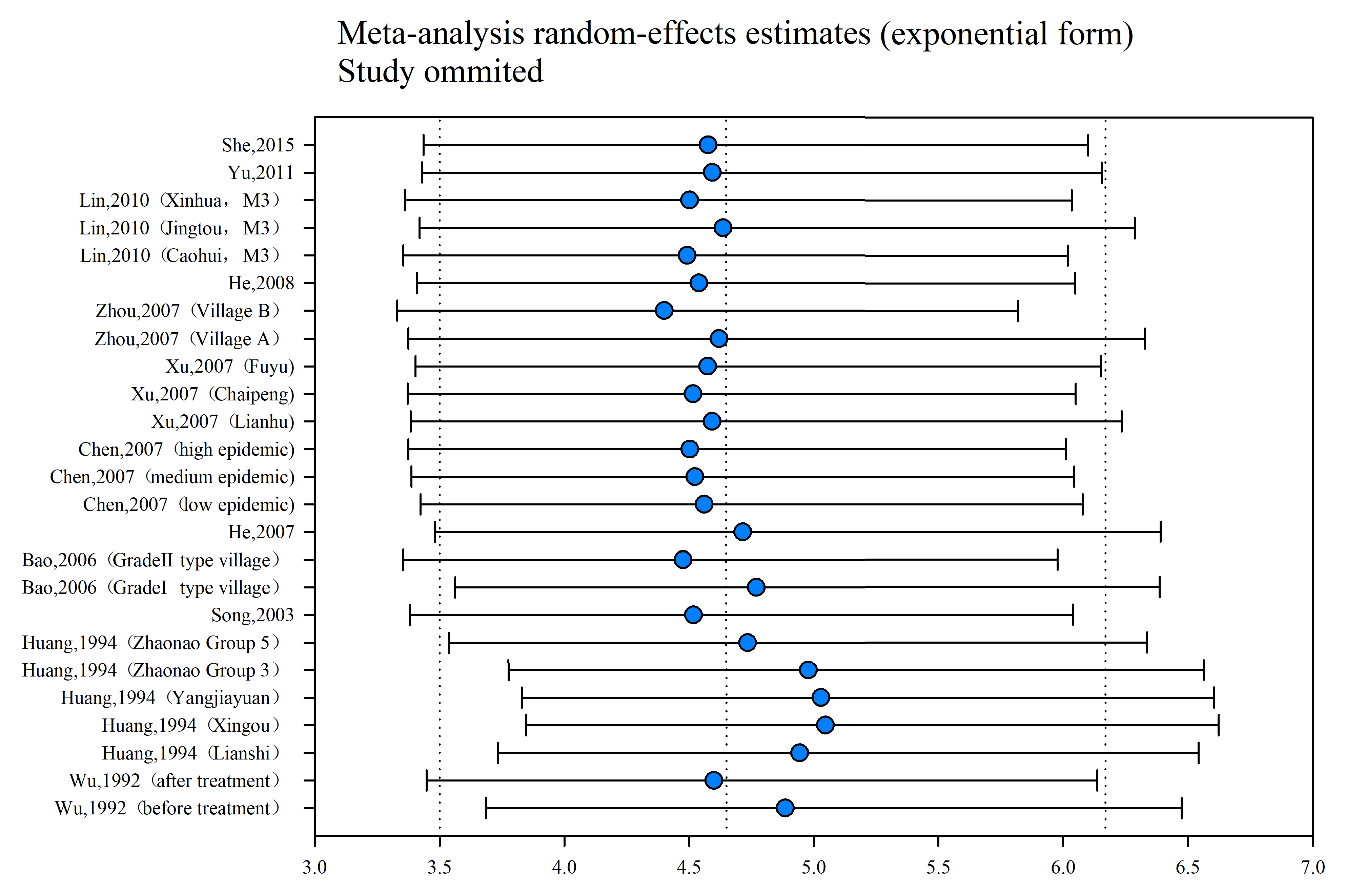

Supplement: Supplementary file 3 — Sensitivity analyses for ELISA to Kato-Katz by single-study-omitted. (TIF 356 kb) [file 12879_2018_3320_MOESM3_ESM.tif]

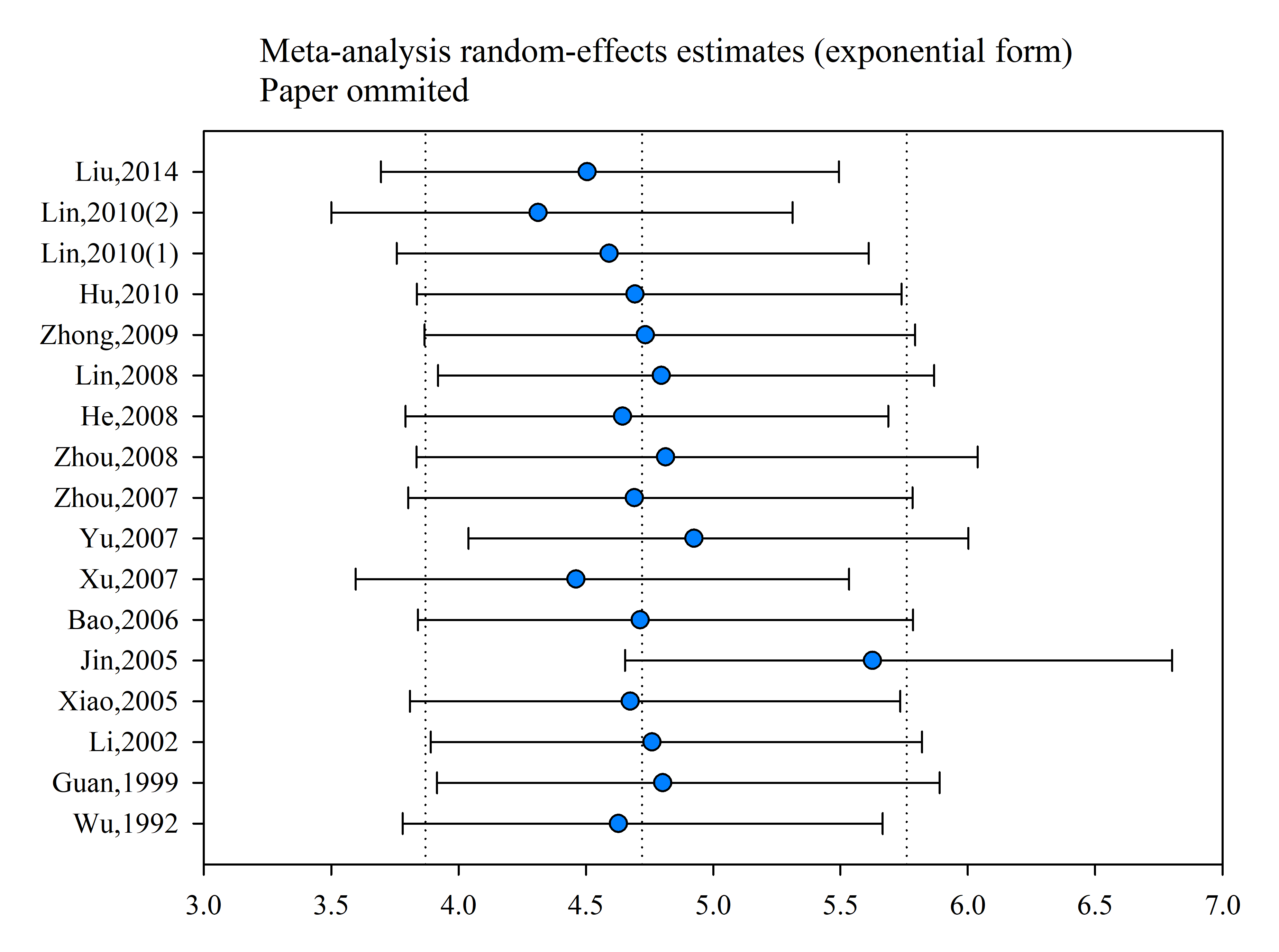

Supplement: Supplementary file 4 — Sensitivity analyses for IHA to Kato-Katz by single-paper-omitted. (TIF 247 kb) [file 12879_2018_3320_MOESM4_ESM.tif]

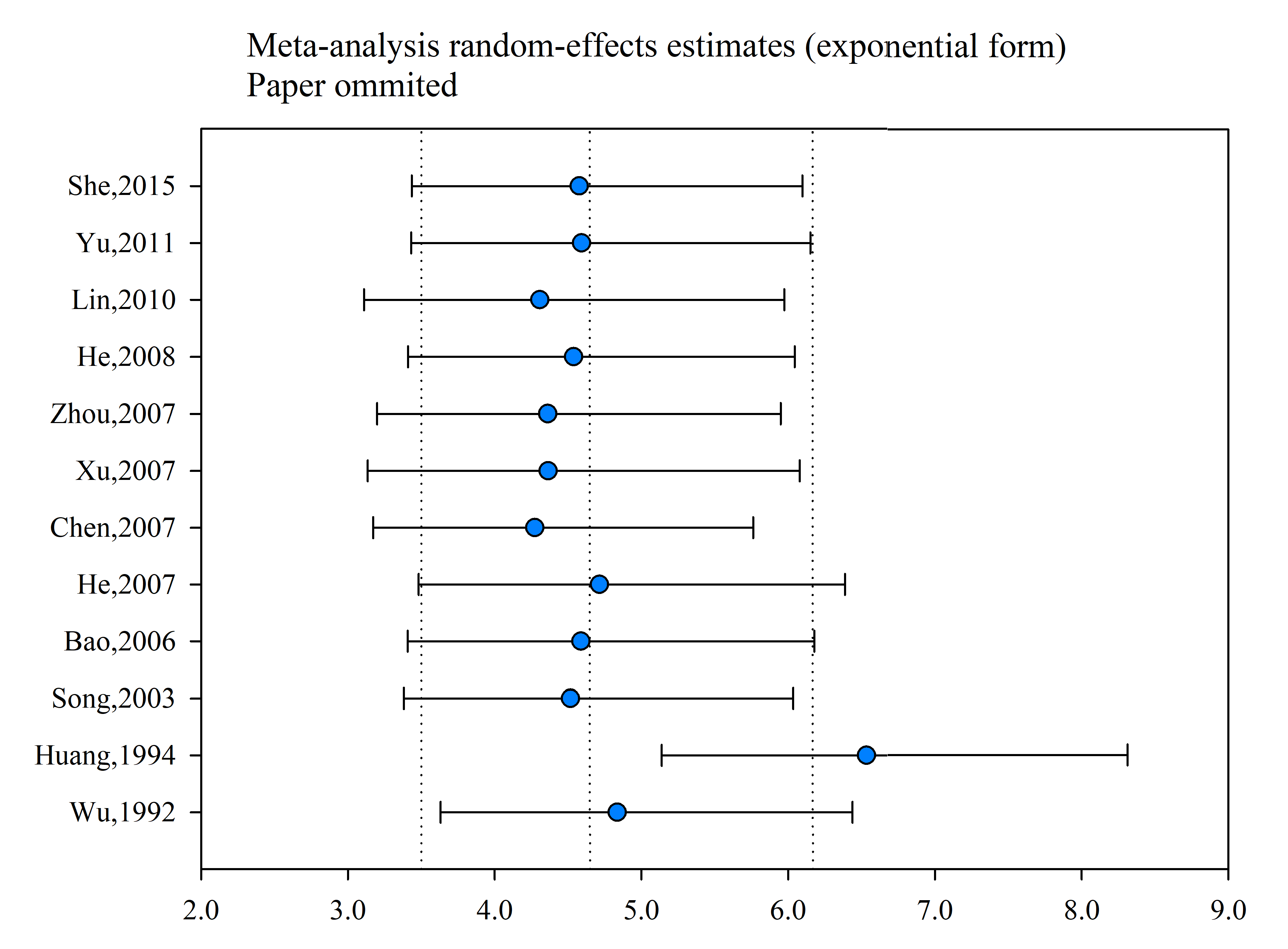

Supplement: Supplementary file 5 — Sensitivity analyses for ELISA to Kato-Katz by single-paper-omitted. (TIF 214 kb) [file 12879_2018_3320_MOESM5_ESM.tif]
